# An iridium complex with an unsupported Ir—Zn bond: di­iodido­(η^5^-penta­methyl­cyclo­penta­dien­yl)bis­(tri­methyl­phosphane)iridiumzinc(*Ir*—*Zn*) benzene hemisolvate

**DOI:** 10.1107/S2056989019014622

**Published:** 2019-11-05

**Authors:** Fioralba Taullaj, Alan J. Lough, Ulrich Fekl

**Affiliations:** a Department of Chemical and Physical Sciences, University of Toronto Mississauga, 3359 Mississauga Rd, Mississauga, Ontario, L5L 1C6, Canada; b Department of Chemistry, University of Toronto, 80 St. George St., Toronto, Ontario, M5S 3H6, Canada

**Keywords:** crystal structure, metal-metal bond, iridium, zinc

## Abstract

The title compound, [IrZnI_2_(C_10_H_15_)(C_3_H_9_P)_2_]·0.5C_6_H_6_ or [Cp*(PMe_3_)_2_Ir]-[ZnI_2_] (Cp* = *cyclo*-C_5_Me_5_) was obtained and characterized as its benzene solvate [Cp*(PMe_3_)_2_Ir]-[ZnI_2_]·0.5C_6_H_6_. The bimetallic complex in this structure contains the Lewis-acidic fragment ZnI_2_ bonded to the Lewis-basic fragment Cp*(PMe_3_)_2_Ir, with an Ir—Zn bond distance of 2.452 (1) Å. The compound was obtained by reacting [Cp*(PMe_3_)IrI_2_] with 2-Ad_2_Zn (2-Ad = 2-adamant­yl), resulting in the reduction of the Ir^III^ complex and formation of the Ir^I^–Zn^II^ adduct. The crystal studied was a twin by non-merohedry with a refined BASF parameter of 0.223 (1).

## Abstract 2

A mol­ecular compound with an unsupported Ir—Zn bond: [Cp*(PMe_3_)_2_Ir]-[ZnI_2_] (Cp*=*cyclo*-C_5_Me_5_), is reported as its benzene solvate.

## Chemical context   

An intuitive way to create metal–metal bonds is by linking a Lewis-basic metal center to a Lewis-acidic metal center. Lewis acid/base adducts of the type [Cp^*R*^(*L*)(*L*′)Ir]-[Zn*X*_2_] (Cp^*R*^ = either Cp, cyclo­penta­dienyl, or Cp*, penta­methyl­cyclo­penta­dienyl; *L* and *L*′ = neutral ligand; *X* = halogen) have been known for a long time. Regarding the Lewis-basic fragment, it has been noted that electron-rich half-sandwich complexes can be considered ‘metal bases *par excellence*’ (Werner, 1983 ▸), and zinc dihalides are among the most well-known Lewis acids. The bimetallic complex [Cp(PPh_3_)(CO)Ir]-[ZnBr_2_] was isolated and spectroscopically characterized 49 years ago (Oliver & Graham, 1970 ▸). However, crystallographic characterization of such complexes having iridium–zinc bonds is elusive. While a related complex [Cp*(CO)_2_Ir]-[ZnCl_2_] was later prepared in a different group, it too was not structurally characterized, instead an adduct with mercury(II) chloride was crystallographically characterized (Einstein *et al.*, 1992 ▸). A cobalt complex [Cp(PMe_3_)_2_Co]-[ZnCl_2_PMe_3_] is known as well; it too is lacking crystallographic characterization (Dey & Werner, 1977 ▸). In fact, while complexes are known where a zinc dihalide acts as a bridge between metals (iridium: Kimura *et al.*, 2012 ▸) or where aggregation occurs to form multi-zinc clusters (rhodium and zinc: Molon *et al.*, 2010 ▸), a search of the Cambridge Crystallographic Database (Groom *et al.*, 2016 ▸) revealed no example of a structurally characterized complex [Cp^*R*^(*L*)(*L*′)*M*]-[Zn*X*_2_] (*M* = either Co, Rh, or Ir) with a terminal (non-bridging) zinc dihalide. For iridium, it appears, in fact, that regardless of the ligand coordination sphere there is no single example of an unsupported iridum–zinc bond. The scarcity of examples for iridium contrasts with the situation of rhodium, for which a couple of examples of unsupported Rh–Zn*X*_2_ structures exist with a PNP ‘pincer’ providing the coordination environment at rhodium (Gair *et al.*, 2019 ▸). Additionally, several Rh–Zn structures exist with Zn–Cp* and Zn–C environments (Cadenbach *et al.*, 2009 ▸). In this contribution, we provide crystallographic characterization for [Cp*(PMe_3_)_2_Ir]-[ZnI_2_] (benzene solvate). The bimetallic complex in this structure is the formal adduct of the Lewis base Cp*(PMe_3_)_2_Ir^I^ and the Lewis acid Zn^II^I_2_, providing the first structural characterization within the large class of metal–metal-bonded compounds [Cp^*R*^(*L*)(*L*′)*M*]-[Zn*X*_2_] (*M* = Co, Rh, or Ir, *X* = halide, *L*, *L*′ = neutral ligand). We did not synthesize the title compound from iridium(I). Rather, it was obtained through the reduction of iridium(III) with di-(2-adamant­yl)zinc, as described under ’*Synthesis and crystallization*’.
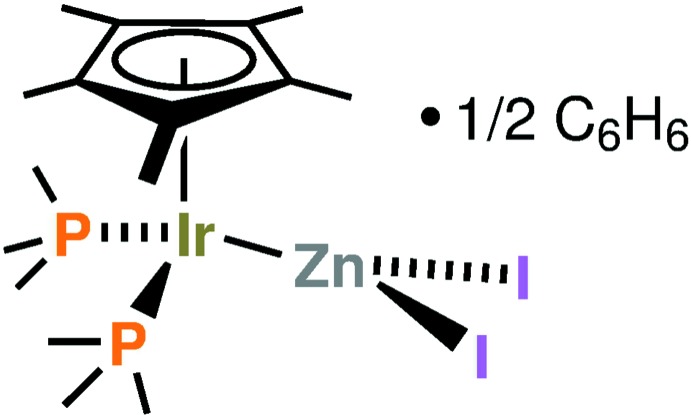


## Structural commentary   

**Figure 1:**
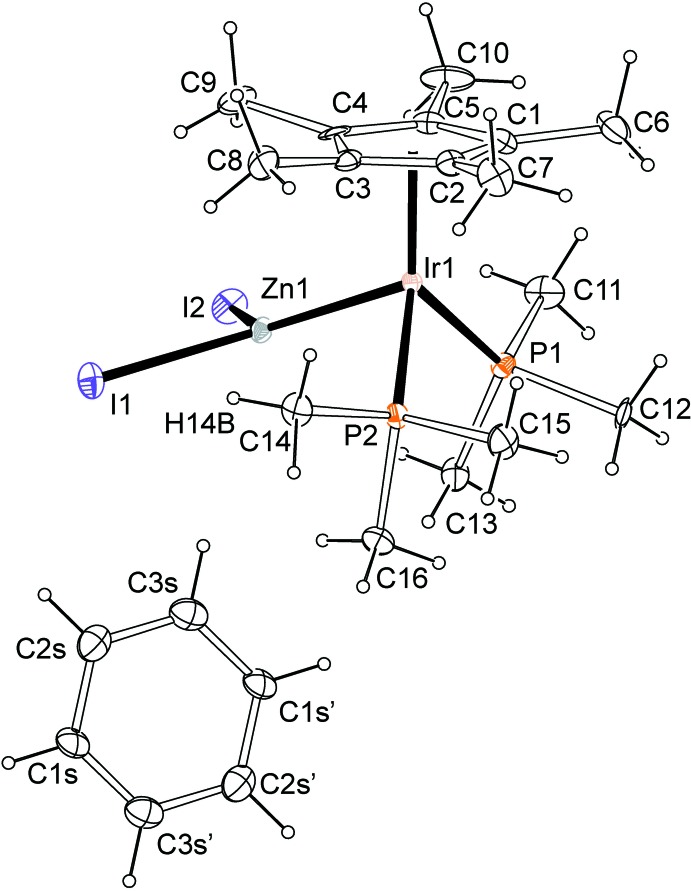
A view of the mol­ecular structure of [Cp*(PMe_3_)_2_Ir]-[ZnI_2_] and its benzene solvate mol­ecule. Anisotropic displacement ellipsoids in this plot, generated with *ORTEP-3 for Windows* (Farrugia, 2012 ▸), are shown at the 30% level. The benzene mol­ecule lies on a crystallographic twofold axis – atoms bearing primed labels are generated by symmetry.

An anisotropic displacement plot showing [Cp*(PMe_3_)_2_Ir]-[ZnI_2_] and its benzene solvate mol­ecule is shown in Fig. 1 ▸. The Ir1—Zn1 distance is 2.452 (1) Å, which is within the expected distance range when compared to other examples of *M*—Zn bonds, specifically those of unsupported Rh—Zn bonds, which were determined to be 2.4224 (6) Å for Rh—ZnCl_2_ and 2.4147 (5) Å for Rh—ZnBr_2_ (Gair *et al.*, 2019 ▸). The only crystallographically characterized Ir—Zn bonds are those of a structure that contains a bridging zinc dihalide, which leads to expected longer *M*—Zn bond distances of 2.563 (1) and 2.566 (1) Å (Kimura *et al.*, 2012 ▸). Bond angles around the iridium center in [Cp*(PMe_3_)_2_Ir]-[ZnI_2_] match those of a three-legged piano stool, with roughly 90° angles. The Zn1—Ir1—P1 angle was found to be 88.74 (7)°, the Zn1—Ir1—P2 angle 91.35 (7)°, and the P1—Ir1—P2 angle 93.81 (9)°. The ZnI_2_ fragment is close to planar, with Zn1 being displaced from the I1–I2–Ir1 plane by only 0.1427 (11) Å. The Zn1—I1 distance is 2.588 (1) Å and the Zn1—I2 distance is 2.582 (1) Å. The angles about Zn are 127.19 (5)° for Ir1—Zn—I1, 126.73 (5)° for Ir1—Zn1—I2, and 105.11 (4)° for I1—Zn1—I2. The larger Ir—Zn—*X* angles and the comparably small *X*—Zn—*X* angle are consistent with what has been observed for the ZnBr_2_ and ZnCl_2_ fragments in the existing Rh–Zn complexes (Gair *et al.*, 2019 ▸). These complexes had Rh1—Zn1—*X*1 (where *X* = Br or Cl) angles of 130.14 (2) and 130.26 (4)°, Rh1—Zn1—*X*2 angles of 120.42 (2) and 120.31 (11)°, and *X*1—Zn1—X2 angles of 109.43 (2) and 109.41 (4)°. In [Cp*(PMe_3_)_2_Ir]-[ZnI_2_], there is a relatively short intra­molecular C—H⋯I inter­action between H14*B* (on the C14 methyl group) and I1, with an H⋯I contact distance of 3.06 Å (this reported distance is based on the calculated position of H14*B*, which is placed at 0.98 Å from C14 and at an angle C14—H14*B*⋯I1 of 157°); the C14⋯I1 distance is 3.977 (12) Å.

## Supra­molecular features   

**Figure 2:**
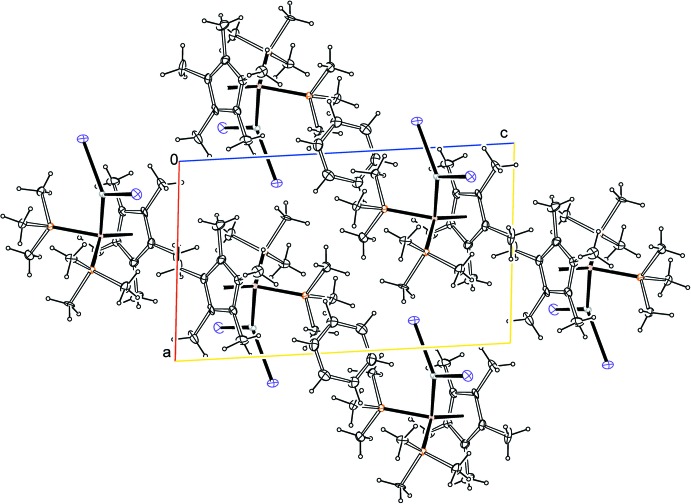
Packing of mol­ecules of [Cp*(PMe_3_)_2_Ir]-[ZnI_2_] and benzene solvate mol­ecules, viewed along the *b* axis.

The packing of [Cp*(PMe_3_)_2_Ir]-[ZnI_2_]·0.5C_6_H_6_ is shown in Fig. 2 ▸. Mol­ecules of [Cp*(PMe_3_)_2_Ir]-[ZnI_2_] and the C_6_H_6_ solvent pack through contacting van der Waals surfaces, without any particular short contacts. There are no inter­molecular hydrogen bonds in the structure. A possible intra­molecular C—H⋯I hydrogen bond is discussed above under *Structural commentary*.

## Database survey   

The Cambridge Crystallographic Database (version 5.40, including updates up to May 2019; Groom *et al.*, 2016 ▸) was searched. No example of an unsupported iridium–zinc bond was found, using the substructure Ir—Zn (any bond). Only one structure was found, namely a structure that contains a bridging zinc dihalide, as discussed under *Chemical context* (Kimura *et al.*, 2012 ▸).

## Synthesis and crystallization   

The synthesis was performed using air-free conditions, solvents were dried over Na/benzo­phenone, [Cp*IrI_2_]_2_ was purchased from Sigma Aldrich, 2-Ad_2_Zn was synthesized according to literature (Armstrong *et al.*, 2017 ▸). [Cp*(PMe_3_)_2_Ir]-[ZnI_2_] was obtained *via* reduction of Cp*(PMe_3_)IrI_2_ with 2-Ad_2_Zn. Cp*(PMe_3_)IrI_2_ was generated *in situ via* reaction of 50mg of [Cp*IrI_2_]_2_ (0.04 mmol) with two equivalents of PMe_3_ (added as a 1 *M* PMe_3_ solution in THF, 100 µL, 0.1 mmol) over 1 h of stirring at room temperature. Next, 30 mg (0.08 mmol) of 2-Ad_2_Zn were added to the reaction mixture, and the reaction was allowed to proceed overnight with stirring at room temperature, resulting in a yellow solution and yellow precipitate. The solution layer was deca­nted into a round-bottom flask, and dried *in vacuo* to yield a yellow solid, which was extracted with C_6_H_6_ forming a colorless solution, with some precipitate forming over time. The colorless crystals of [Cp*(PMe_3_)_2_Ir]-[ZnI_2_] grew out of the benzene solution *via* slow evaporation at room temperature.

## Refinement   

Crystal data, data collection and structure refinement details are summarized in Table 1 ▸. The crystal studied was a twin by non-merohedry with a twin transformation matrix of 1.00 0.00 0.00, −0.90 − 1.00 0.00, 0.06 0.00 − 1.00 and a refined BASF parameter of 0.223 (1). The *TWINABS* (Bruker, 2012 ▸) function in *APEX2* (Bruker, 2014 ▸) was used to de-twin the data. *U^ij^* components of ADPs for atoms C1 through C5 were restrained to be similar to each other (SIMU 0.01 command of *SHELXL*).

## Supplementary Material

Crystal structure: contains datablock(s) I. DOI: 10.1107/S2056989019014622/zl2763sup1.cif

Structure factors: contains datablock(s) I. DOI: 10.1107/S2056989019014622/zl2763Isup2.hkl

CCDC references: 1961812, 1961812

Additional supporting information:  crystallographic information; 3D view; checkCIF report

## Figures and Tables

**Table 1:** Experimental details

Crystal data	
Chemical formula	[IrZnI_2_(C_10_H_15_)(C_3_H_9_P)_2_]·0.5C_6_H_6_
*M*_r_	837.79
Crystal system, space group	Triclinic, *P* 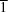
Temperature (K)	150
*a*, *b*, *c* (Å)	9.5353 (7), 10.1962 (8), 14.6331 (10)
α, β, γ (°)	95.975 (2), 91.255 (2), 114.847 (2)
*V* (Å^3^)	1280.58 (16)
*Z*	2
Radiation type	Mo *K*α
μ (mm^−1^)	8.67
Crystal size (mm)	0.22 × 0.03 × 0.02

Data collection	
Diffractometer	Bruker Kappa *APEX* DUO CCD
Absorption correction	Multi-scan (*TWINABS*; Bruker, 2012 ▸)
*T*_min_, *T*_max_	0.560, 0.746
No. of measured, independent and observed [*I* > 2σ(*I*)] reflections	56694, 5865, 4967
*R*_int_	0.056
(sin θ/λ)_max_ (Å^−1^)	0.651

Refinement	
*R*[*F* ^2^ > 2σ(*F* ^2^)], *wR*(*F* ^2^), *S*	0.040, 0.116, 1.13
No. of reflections	5865
No. of parameters	238
No. of restraints	30
H-atom treatment	H-atom parameters constrained
Δρ_max_, Δρ_min_ (e Å^−3^)	1.81, −1.66

Computer programs: *APEX2* and *SAINT* (Bruker, 2014 ▸), *SHELXT* (Sheldrick, 2015*a* ▸), *SHELXL2016* (Sheldrick, 2015*b* ▸), *PLATON* (Spek, 2009 ▸) and *SHELXTL* (Sheldrick, 2008 ▸).

## supplementary crystallographic information

### Crystal data

**Table:**

[IrZnI_2_(C_10_H_15_)(C_3_H_9_P)_2_]·0.5C_6_H_6_	*Z* = 2
*M**_r_* = 837.79	*F*(000) = 786
Triclinic, *P*1	*D*_x_ = 2.173 Mg m^−^^3^
*a* = 9.5353 (7) Å	Mo *K*α radiation, λ = 0.71073 Å
*b* = 10.1962 (8) Å	Cell parameters from 9876 reflections
*c* = 14.6331 (10) Å	θ = 2.2–27.5°
α = 95.975 (2)°	µ = 8.66 mm^−^^1^
β = 91.255 (2)°	*T* = 150 K
γ = 114.847 (2)°	Needle, colourless
*V* = 1280.58 (16) Å^3^	0.22 × 0.03 × 0.02 mm

### Data collection

**Table:**

Bruker Kappa APEX DUO CCD diffractometer	4967 reflections with *I* > 2σ(*I*)
Radiation source: sealed tube with Bruker Triumph monochromator	*R*_int_ = 0.056
φ and ω scans	θ_max_ = 27.6°, θ_min_ = 1.4°
Absorption correction: multi-scan (*TWINABS*; Bruker, 2012)	*h* = −12→12
*T*_min_ = 0.560, *T*_max_ = 0.746	*k* = −13→13
56694 measured reflections	*l* = 0→19
5865 independent reflections	

### Refinement

**Table:**

Refinement on *F*^2^	Primary atom site location: structure-invariant direct methods
Least-squares matrix: full	Hydrogen site location: inferred from neighbouring sites
*R*[*F*^2^ > 2σ(*F*^2^)] = 0.040	H-atom parameters constrained
*wR*(*F*^2^) = 0.116	*w* = 1/[σ^2^(*F*_o_^2^) + (0.0542*P*)^2^ + 11.6185*P*] where *P* = (*F*_o_^2^ + 2*F*_c_^2^)/3
*S* = 1.13	(Δ/σ)_max_ = 0.001
5865 reflections	Δρ_max_ = 1.81 e Å^−^^3^
238 parameters	Δρ_min_ = −1.66 e Å^−^^3^
30 restraints	

### Special details

**Table:**

Geometry. All esds (except the esd in the dihedral angle between two l.s. planes) are estimated using the full covariance matrix. The cell esds are taken into account individually in the estimation of esds in distances, angles and torsion angles; correlations between esds in cell parameters are only used when they are defined by crystal symmetry. An approximate (isotropic) treatment of cell esds is used for estimating esds involving l.s. planes.
Refinement. Refined as a 2-component twin

### Fractional atomic coordinates and isotropic or equivalent isotropic displacement parameters (Å^2^)

**Table:**

	*x*	*y*	*z*	*U*_iso_*/*U*_eq_	
Ir1	0.64620 (4)	0.30438 (4)	0.23280 (2)	0.01416 (10)	
Zn1	0.85316 (12)	0.54793 (12)	0.22619 (7)	0.0182 (2)	
I1	1.13849 (8)	0.64673 (8)	0.29085 (5)	0.03107 (17)	
I2	0.84656 (9)	0.74030 (8)	0.12668 (5)	0.03387 (18)	
P1	0.4792 (3)	0.4076 (3)	0.25762 (17)	0.0184 (5)	
P2	0.7072 (3)	0.3167 (3)	0.38389 (16)	0.0184 (5)	
C1	0.5046 (12)	0.1007 (11)	0.1315 (7)	0.028 (2)	
C2	0.6108 (11)	0.0698 (10)	0.1844 (7)	0.0216 (18)	
C3	0.7631 (12)	0.1679 (11)	0.1690 (7)	0.0224 (18)	
C4	0.7521 (13)	0.2568 (12)	0.1035 (7)	0.027 (2)	
C5	0.5942 (13)	0.2200 (11)	0.0809 (6)	0.0260 (19)	
C6	0.3309 (13)	0.0135 (13)	0.1213 (9)	0.039 (3)	
H6A	0.302415	−0.058383	0.066236	0.059*	
H6B	0.280848	0.079058	0.115315	0.059*	
H6C	0.296607	−0.036785	0.175766	0.059*	
C7	0.5730 (14)	−0.0549 (12)	0.2404 (9)	0.036 (3)	
H7A	0.587149	−0.134379	0.204256	0.054*	
H7B	0.465243	−0.089402	0.256554	0.054*	
H7C	0.642101	−0.022047	0.296775	0.054*	
C8	0.9134 (13)	0.1610 (14)	0.1988 (9)	0.038 (3)	
H8A	0.951864	0.122296	0.145845	0.056*	
H8B	0.894569	0.097313	0.247218	0.056*	
H8C	0.990738	0.259090	0.222445	0.056*	
C9	0.8854 (16)	0.3580 (13)	0.0514 (8)	0.042 (3)	
H9A	0.891465	0.304718	−0.007005	0.064*	
H9B	0.983291	0.391807	0.088981	0.064*	
H9C	0.866425	0.442011	0.038967	0.064*	
C10	0.5338 (18)	0.2725 (17)	0.0030 (8)	0.049 (4)	
H10A	0.561866	0.237987	−0.055749	0.074*	
H10B	0.579434	0.379230	0.011319	0.074*	
H10C	0.420718	0.234441	0.002733	0.074*	
C11	0.3809 (14)	0.4250 (14)	0.1551 (8)	0.035 (3)	
H11A	0.305145	0.462747	0.173110	0.053*	
H11B	0.327569	0.329362	0.118423	0.053*	
H11C	0.457013	0.492346	0.118469	0.053*	
C12	0.3114 (11)	0.3065 (12)	0.3218 (8)	0.026 (2)	
H12A	0.237710	0.350330	0.319995	0.040*	
H12B	0.346343	0.310499	0.385961	0.040*	
H12C	0.260818	0.204706	0.293688	0.040*	
C13	0.5420 (12)	0.5912 (11)	0.3179 (8)	0.028 (2)	
H13A	0.451169	0.609824	0.330705	0.042*	
H13B	0.608472	0.662236	0.279284	0.042*	
H13C	0.600217	0.600006	0.375988	0.042*	
C14	0.8854 (12)	0.2999 (13)	0.4124 (7)	0.027 (2)	
H14A	0.905137	0.313444	0.479545	0.041*	
H14B	0.972012	0.374360	0.385704	0.041*	
H14C	0.874887	0.203020	0.387452	0.041*	
C15	0.5697 (13)	0.1720 (12)	0.4447 (7)	0.034 (3)	
H15A	0.609739	0.184805	0.508784	0.051*	
H15B	0.556651	0.076787	0.414398	0.051*	
H15C	0.469393	0.177272	0.443155	0.051*	
C16	0.7304 (13)	0.4736 (11)	0.4657 (7)	0.029 (2)	
H16A	0.772321	0.465670	0.525593	0.043*	
H16B	0.629520	0.476117	0.472535	0.043*	
H16C	0.801849	0.563231	0.442954	0.043*	
C1S	1.1540 (14)	1.0646 (12)	0.5329 (8)	0.033 (3)	
H1S	1.259917	1.108754	0.555309	0.040*	
C2S	1.1106 (14)	0.9857 (13)	0.4464 (9)	0.037 (3)	
H2S	1.186666	0.975586	0.409107	0.045*	
C3S	0.9552 (14)	0.9211 (13)	0.4139 (8)	0.037 (3)	
H3S	0.925314	0.866769	0.354327	0.045*	

### Atomic displacement parameters (Å^2^)

**Table:**

	*U*^11^	*U*^22^	*U*^33^	*U*^12^	*U*^13^	*U*^23^
Ir1	0.01386 (16)	0.01348 (16)	0.01504 (16)	0.00549 (13)	0.00113 (12)	0.00274 (11)
Zn1	0.0172 (5)	0.0147 (5)	0.0226 (5)	0.0060 (4)	0.0056 (4)	0.0042 (4)
I1	0.0205 (3)	0.0307 (4)	0.0406 (4)	0.0092 (3)	0.0000 (3)	0.0061 (3)
I2	0.0403 (4)	0.0313 (4)	0.0366 (4)	0.0197 (3)	0.0079 (3)	0.0114 (3)
P1	0.0175 (12)	0.0213 (12)	0.0192 (12)	0.0108 (10)	0.0015 (9)	0.0038 (9)
P2	0.0168 (11)	0.0198 (12)	0.0172 (11)	0.0058 (9)	0.0004 (9)	0.0057 (9)
C1	0.025 (4)	0.025 (4)	0.026 (4)	0.007 (4)	−0.003 (4)	−0.007 (4)
C2	0.028 (4)	0.014 (4)	0.024 (4)	0.011 (3)	0.003 (3)	0.003 (3)
C3	0.030 (4)	0.018 (4)	0.021 (4)	0.012 (3)	0.007 (3)	−0.002 (3)
C4	0.036 (5)	0.028 (4)	0.015 (4)	0.015 (4)	0.012 (3)	−0.007 (3)
C5	0.035 (4)	0.027 (4)	0.017 (4)	0.016 (4)	−0.001 (3)	−0.003 (3)
C6	0.030 (6)	0.031 (6)	0.052 (8)	0.014 (5)	−0.012 (5)	−0.017 (5)
C7	0.042 (7)	0.022 (6)	0.046 (7)	0.013 (5)	0.000 (5)	0.013 (5)
C8	0.026 (6)	0.036 (7)	0.055 (8)	0.021 (5)	0.005 (5)	−0.007 (6)
C9	0.057 (8)	0.030 (6)	0.034 (7)	0.011 (6)	0.027 (6)	0.004 (5)
C10	0.086 (11)	0.061 (9)	0.016 (6)	0.049 (8)	−0.010 (6)	−0.004 (6)
C11	0.043 (7)	0.044 (7)	0.032 (6)	0.033 (6)	−0.007 (5)	0.003 (5)
C12	0.011 (4)	0.028 (6)	0.037 (6)	0.005 (4)	0.013 (4)	0.005 (4)
C13	0.024 (5)	0.017 (5)	0.045 (7)	0.011 (4)	0.005 (4)	0.003 (4)
C14	0.027 (5)	0.036 (6)	0.026 (5)	0.017 (5)	−0.003 (4)	0.012 (4)
C15	0.033 (6)	0.034 (6)	0.029 (5)	0.006 (5)	0.005 (4)	0.014 (5)
C16	0.031 (6)	0.030 (5)	0.023 (5)	0.012 (5)	−0.004 (4)	0.000 (4)
C1S	0.036 (6)	0.018 (5)	0.041 (7)	0.009 (5)	0.000 (5)	−0.006 (5)
C2S	0.040 (7)	0.026 (6)	0.047 (7)	0.014 (5)	0.015 (5)	0.010 (5)
C3S	0.048 (8)	0.031 (6)	0.030 (6)	0.015 (5)	0.000 (5)	0.004 (4)

### Geometric parameters (Å, º)

**Table:**

Ir1—P2	2.251 (2)	C8—H8B	0.9800
Ir1—C5	2.264 (9)	C8—H8C	0.9800
Ir1—P1	2.265 (2)	C9—H9A	0.9800
Ir1—C3	2.265 (9)	C9—H9B	0.9800
Ir1—C4	2.267 (9)	C9—H9C	0.9800
Ir1—C1	2.291 (10)	C10—H10A	0.9800
Ir1—C2	2.301 (9)	C10—H10B	0.9800
Ir1—Zn1	2.4516 (11)	C10—H10C	0.9800
Zn1—I2	2.5819 (13)	C11—H11A	0.9800
Zn1—I1	2.5880 (13)	C11—H11B	0.9800
P1—C11	1.815 (11)	C11—H11C	0.9800
P1—C13	1.825 (11)	C12—H12A	0.9800
P1—C12	1.840 (10)	C12—H12B	0.9800
P2—C14	1.822 (10)	C12—H12C	0.9800
P2—C16	1.826 (10)	C13—H13A	0.9800
P2—C15	1.840 (10)	C13—H13B	0.9800
C1—C2	1.417 (14)	C13—H13C	0.9800
C1—C5	1.449 (15)	C14—H14A	0.9800
C1—C6	1.510 (15)	C14—H14B	0.9800
C2—C3	1.414 (14)	C14—H14C	0.9800
C2—C7	1.504 (14)	C15—H15A	0.9800
C3—C4	1.417 (15)	C15—H15B	0.9800
C3—C8	1.521 (15)	C15—H15C	0.9800
C4—C5	1.412 (15)	C16—H16A	0.9800
C4—C9	1.538 (15)	C16—H16B	0.9800
C5—C10	1.509 (15)	C16—H16C	0.9800
C6—H6A	0.9800	C1S—C3S^i^	1.360 (17)
C6—H6B	0.9800	C1S—C2S	1.381 (17)
C6—H6C	0.9800	C1S—H1S	0.9500
C7—H7A	0.9800	C2S—C3S	1.393 (18)
C7—H7B	0.9800	C2S—H2S	0.9500
C7—H7C	0.9800	C3S—H3S	0.9500
C8—H8A	0.9800		
			
P2—Ir1—C5	160.3 (3)	C1—C6—H6B	109.5
P2—Ir1—P1	93.81 (9)	H6A—C6—H6B	109.5
C5—Ir1—P1	102.1 (3)	C1—C6—H6C	109.5
P2—Ir1—C3	101.5 (3)	H6A—C6—H6C	109.5
C5—Ir1—C3	61.2 (4)	H6B—C6—H6C	109.5
P1—Ir1—C3	162.4 (3)	C2—C7—H7A	109.5
P2—Ir1—C4	133.0 (3)	C2—C7—H7B	109.5
C5—Ir1—C4	36.3 (4)	H7A—C7—H7B	109.5
P1—Ir1—C4	131.8 (3)	C2—C7—H7C	109.5
C3—Ir1—C4	36.4 (4)	H7A—C7—H7C	109.5
P2—Ir1—C1	127.8 (3)	H7B—C7—H7C	109.5
C5—Ir1—C1	37.1 (4)	C3—C8—H8A	109.5
P1—Ir1—C1	103.0 (3)	C3—C8—H8B	109.5
C3—Ir1—C1	60.6 (4)	H8A—C8—H8B	109.5
C4—Ir1—C1	60.6 (4)	C3—C8—H8C	109.5
P2—Ir1—C2	99.7 (3)	H8A—C8—H8C	109.5
C5—Ir1—C2	60.8 (4)	H8B—C8—H8C	109.5
P1—Ir1—C2	132.8 (3)	C4—C9—H9A	109.5
C3—Ir1—C2	36.1 (4)	C4—C9—H9B	109.5
C4—Ir1—C2	60.1 (4)	H9A—C9—H9B	109.5
C1—Ir1—C2	36.0 (4)	C4—C9—H9C	109.5
P2—Ir1—Zn1	91.35 (7)	H9A—C9—H9C	109.5
C5—Ir1—Zn1	100.5 (3)	H9B—C9—H9C	109.5
P1—Ir1—Zn1	88.74 (7)	C5—C10—H10A	109.5
C3—Ir1—Zn1	99.4 (3)	C5—C10—H10B	109.5
C4—Ir1—Zn1	80.8 (3)	H10A—C10—H10B	109.5
C1—Ir1—Zn1	137.2 (3)	C5—C10—H10C	109.5
C2—Ir1—Zn1	135.3 (2)	H10A—C10—H10C	109.5
Ir1—Zn1—I2	126.73 (5)	H10B—C10—H10C	109.5
Ir1—Zn1—I1	127.19 (5)	P1—C11—H11A	109.5
I2—Zn1—I1	105.11 (4)	P1—C11—H11B	109.5
C11—P1—C13	99.5 (5)	H11A—C11—H11B	109.5
C11—P1—C12	100.2 (6)	P1—C11—H11C	109.5
C13—P1—C12	100.5 (5)	H11A—C11—H11C	109.5
C11—P1—Ir1	115.7 (4)	H11B—C11—H11C	109.5
C13—P1—Ir1	121.9 (3)	P1—C12—H12A	109.5
C12—P1—Ir1	115.6 (4)	P1—C12—H12B	109.5
C14—P2—C16	100.7 (5)	H12A—C12—H12B	109.5
C14—P2—C15	100.1 (5)	P1—C12—H12C	109.5
C16—P2—C15	98.6 (5)	H12A—C12—H12C	109.5
C14—P2—Ir1	115.9 (4)	H12B—C12—H12C	109.5
C16—P2—Ir1	121.8 (3)	P1—C13—H13A	109.5
C15—P2—Ir1	116.2 (4)	P1—C13—H13B	109.5
C2—C1—C5	107.5 (9)	H13A—C13—H13B	109.5
C2—C1—C6	125.9 (10)	P1—C13—H13C	109.5
C5—C1—C6	126.3 (10)	H13A—C13—H13C	109.5
C2—C1—Ir1	72.4 (6)	H13B—C13—H13C	109.5
C5—C1—Ir1	70.4 (5)	P2—C14—H14A	109.5
C6—C1—Ir1	127.9 (8)	P2—C14—H14B	109.5
C3—C2—C1	108.6 (9)	H14A—C14—H14B	109.5
C3—C2—C7	124.2 (9)	P2—C14—H14C	109.5
C1—C2—C7	126.9 (10)	H14A—C14—H14C	109.5
C3—C2—Ir1	70.6 (5)	H14B—C14—H14C	109.5
C1—C2—Ir1	71.7 (6)	P2—C15—H15A	109.5
C7—C2—Ir1	128.4 (8)	P2—C15—H15B	109.5
C2—C3—C4	107.8 (9)	H15A—C15—H15B	109.5
C2—C3—C8	127.2 (10)	P2—C15—H15C	109.5
C4—C3—C8	123.8 (10)	H15A—C15—H15C	109.5
C2—C3—Ir1	73.3 (5)	H15B—C15—H15C	109.5
C4—C3—Ir1	71.9 (5)	P2—C16—H16A	109.5
C8—C3—Ir1	130.4 (7)	P2—C16—H16B	109.5
C5—C4—C3	109.0 (9)	H16A—C16—H16B	109.5
C5—C4—C9	123.7 (11)	P2—C16—H16C	109.5
C3—C4—C9	126.4 (10)	H16A—C16—H16C	109.5
C5—C4—Ir1	71.7 (5)	H16B—C16—H16C	109.5
C3—C4—Ir1	71.7 (5)	C3S^i^—C1S—C2S	119.9 (11)
C9—C4—Ir1	130.9 (7)	C3S^i^—C1S—H1S	120.0
C4—C5—C1	107.0 (9)	C2S—C1S—H1S	120.0
C4—C5—C10	125.0 (11)	C1S—C2S—C3S	119.9 (11)
C1—C5—C10	126.6 (11)	C1S—C2S—H2S	120.1
C4—C5—Ir1	72.0 (5)	C3S—C2S—H2S	120.1
C1—C5—Ir1	72.5 (5)	C1S^i^—C3S—C2S	120.2 (11)
C10—C5—Ir1	131.1 (8)	C1S^i^—C3S—H3S	119.9
C1—C6—H6A	109.5	C2S—C3S—H3S	119.9
			
C5—C1—C2—C3	0.8 (11)	Ir1—C3—C4—C9	127.8 (10)
C6—C1—C2—C3	174.3 (10)	C2—C3—C4—Ir1	65.0 (7)
Ir1—C1—C2—C3	−61.2 (7)	C8—C3—C4—Ir1	−127.0 (10)
C5—C1—C2—C7	−173.2 (10)	C3—C4—C5—C1	−2.1 (10)
C6—C1—C2—C7	0.3 (17)	C9—C4—C5—C1	168.1 (10)
Ir1—C1—C2—C7	124.7 (11)	Ir1—C4—C5—C1	−64.5 (7)
C5—C1—C2—Ir1	62.1 (7)	C3—C4—C5—C10	−169.6 (10)
C6—C1—C2—Ir1	−124.5 (11)	C9—C4—C5—C10	0.6 (15)
C1—C2—C3—C4	−2.1 (11)	Ir1—C4—C5—C10	128.0 (10)
C7—C2—C3—C4	172.1 (10)	C3—C4—C5—Ir1	62.4 (6)
Ir1—C2—C3—C4	−64.1 (7)	C9—C4—C5—Ir1	−127.4 (10)
C1—C2—C3—C8	−169.6 (10)	C2—C1—C5—C4	0.8 (11)
C7—C2—C3—C8	4.6 (16)	C6—C1—C5—C4	−172.6 (10)
Ir1—C2—C3—C8	128.5 (10)	Ir1—C1—C5—C4	64.2 (6)
C1—C2—C3—Ir1	61.9 (7)	C2—C1—C5—C10	168.1 (10)
C7—C2—C3—Ir1	−123.9 (10)	C6—C1—C5—C10	−5.4 (17)
C2—C3—C4—C5	2.7 (11)	Ir1—C1—C5—C10	−128.6 (11)
C8—C3—C4—C5	170.7 (9)	C2—C1—C5—Ir1	−63.4 (7)
Ir1—C3—C4—C5	−62.4 (6)	C6—C1—C5—Ir1	123.2 (11)
C2—C3—C4—C9	−167.2 (10)	C3S^i^—C1S—C2S—C3S	0 (2)
C8—C3—C4—C9	0.8 (16)	C1S—C2S—C3S—C1S^i^	0 (2)

Symmetry code: (i) −*x*+2, −*y*+2, −*z*+1.
